# Psychiatry’s role in the prevention of post-intensive care mental health impairment: stakeholder survey

**DOI:** 10.1186/s12888-022-03855-w

**Published:** 2022-03-18

**Authors:** Ewa D. Bieber, Kemuel L. Philbrick, Jenna B. Shapiro, Lioudmila V. Karnatovskaia

**Affiliations:** 1 Department of Psychiatry and Psychology, Mayo Clinic, Rochester, MN USA; 2 Department of Psychiatry and Behavioral Health, Ann and Robert H. Lurie Children’s Hospital of Chicago, Chicago, IL USA; 3 Department of Psychiatry and Behavioral Sciences, Northwestern University Feinberg School of Medicine, Chicago, IL USA; 4 Department of Medicine, Mayo Clinic, 200 First St SW, Rochester, MN 55905 USA

**Keywords:** Critical illness, Mental health, Post-traumatic, Depression, Patient safety

## Abstract

**Background:**

Many critical illness survivors experience new or worsening mental health impairments. Psychiatry consultation services can provide a critical role in identifying, addressing, and preventing mental health challenges during and after admission to the acute medical care setting. However, psychiatry involvement in the ICU setting is lower than in other hospital settings and the conventional process in many hospitals requires other care providers to request consultation by psychiatry. Despite these differences, no studies have sought ICU provider perspectives on psychiatry consultation’s current and desired role. We aimed to obtain stakeholder feedback on psychiatry’s current and desired roles in the ICU, and potential benefits and drawbacks of increasing psychiatry’s presence.

**Methods:**

A web-based survey obtained perspectives from 373 critical care physicians and advance practice providers, bedside nurses, physical and occupational therapists, pharmacists, and consultation-liaison psychiatry physicians and advance practice providers at a tertiary care center using multiple choice and open-ended questions. Descriptive information and content analysis of qualitative data provided information on stakeholder perspectives.

**Results:**

Psychiatry’s primary current role was seen as assistance with management of mental health issues (38%) and suicide risk assessments (23%). 46% wished for psychiatry’s increased involvement in the ICU. Perceived benefits of increased psychiatry presence in the ICU included early psychological support in parallel with medical care, identification of psychiatric factors impacting treatment, and facilitation of family understanding of the patient’s mental state/delirium. An additional perceived benefit included reduction in provider burnout through processing difficult situations and decreasing family psychological distress. However, one concern included potential conflict among providers regarding treatment.

**Conclusions:**

Those who work closely with the critically ill patients think that increased psychological support in the ICU would be beneficial. By contrast, psychiatry’s current involvement is seen to be limited, perhaps driven by varying perceptions of what psychiatry’s role is or should be.

**Supplementary Information:**

The online version contains supplementary material available at 10.1186/s12888-022-03855-w.

## Background

Approximately 6 million people in the United States are hospitalized in an intensive care unit (ICU) annually for a variety of life-threatening problems [1]. Due to ongoing advancements in medical technology and knowledge, the majority of patients survive to discharge; [2] however, a significant percentage experience new or worsening physical, cognitive and/or mental health impairments, collectively termed Post Intensive Care Syndrome (PICS) [3]. Mental health impairments include symptoms of anxiety, depression and post-traumatic stress. Treatment has proven to be challenging, with many impairments becoming chronic, resulting in significant quality of life impairment, negative impact on future medical treatment, and increased health care utilization [4–7].

Several risk factors for the development of PICS have been identified, among them a pre-existing psychiatric illness, [8] which afflicts up to 25% of patients admitted to the ICU [9]. Previous studies have suggested that identification of patients with preexisting mental health morbidity may help minimize PICS through prevention and early intervention efforts [7]. Amelioration of PICS symptoms is a multidisciplinary attempt with interventions such as nursing-led ICU diaries, early mobilization by physical therapy, cognitive interventions with occupational therapy, and medication adjustments monitored by pharmacist [10]. Psychiatry consultation in the ICU may add to these efforts through identification of pre-existing psychiatric concerns and delirium, provision of recommendations to address and prevent mental health concerns while in the ICU and post-discharge. However, requests for psychiatric consultation from the ICU are rare compared to other medical units [11]. The reasons behind this lower rate of psychiatric consultation remain unclear.

One recent systematic review of psychiatric consultation by other non-ICU hospital services identified physician-reported barriers to consultation that include concern for stigma, belief that another mental health professional or the medical team may address psychiatric concerns equally well, poor rapport between the medical team and the psychiatrist, and poor recognition of mental illness [12]. However, no studies examining ICU provider perceptions of psychiatry’s current and desired roles in the ICU have been published to date despite heightened concern for elevated psychiatric risk during and after ICU admission. As such, it is not clear if similar perspectives on psychiatry’s role and potential barriers to referral are shared in the more acute ICU setting.

To bridge this gap and inform ICU psychiatric consultation practices, this study aimed to evaluate ICU stakeholders’ perspectives in four domains: psychiatry’s current role in the ICU, the desired role of psychiatry in the ICU, the perceived benefit of increasing psychiatry’s presence in the ICU, and barriers to or drawbacks of increasing psychiatry’s presence in the ICU. Based on previous literature, it was hypothesized that psychiatry’s current involvement would be perceived as limited, that there would be high desire for increased collaboration with current potential barriers to implementation noted, and few drawbacks to greater collaboration would be identified. Information from the current study was expected to be helpful for guiding refinement of the current psychiatric consultation model, enhancing collaboration between the teams caring for ICU patients, and moving further toward an eventual goal of improving mental health outcomes for ICU patients during admission and following discharge.

## Methods

The current study collected survey data from critical care physicians, trainees, and advance practice providers bedside nurses, physical and occupational therapists, and pharmacists across several ICU settings at a large, tertiary center in Minnesota, United States. Psychiatry physicians, trainees and advance practice providers who provide psychiatric consultations to these ICUs in a conventional referral-based, rather than proactive, model were also surveyed. The psychiatry consultation service does not include a psychologist nor is there a separate psychology consultation service. The participants are collectively referred to as “ICU stakeholders”.

The survey was reviewed by the Mayo clinic institutional review board (IRB) and was deemed as not needing an IRB approval. As such, informed consent was not required, and a response to the survey constituted agreement to participate in the study. Participants were notified that responding was completely voluntary and would not impact employment.

### Sample and procedure

Questionnaires were sent using the REDCap online survey database (Nashville, 2020) to 1039 ICU stakeholders working across two medical, two surgical, and one combined medical/surgical ICU. Potential participants were identified through their respective department’s email distribution list. Each participant received a unique link which was de-activated once that individual’s survey was completed. A reminder email was re-sent to those who did not respond after one week. Data were collected between January 16 and January 23, 2020. Participants completed the survey anonymously and data were not linked to any identifying information.

### Questionnaires

Surveys included twelve questions with multiple choice and open-ended formats, which were developed by EDB, LVK, and KLP. Questions were formulated based on prior literature to expand insight into previously published dynamics on psychiatry consultation in hospital settings [12]. Participants were asked to identify their professional role, primary work setting, and the frequency with which they professionally interact with psychiatry, their understanding of ICU patients’ experiences and the best approach to prevent PICS. Multiple choice and open-ended questions were also used to obtain information on participants’ perspective of psychiatry’s current primary role in the ICU, potential benefit of psychiatry’s increased presence in the ICU, potential barriers and drawbacks to increased presence, and the desired level of involvement of psychiatry in the ICU (questionnaire included as Supplemental Table 1). Multiple choice options were selected based on review of prior literature in non-ICU settings with adaptations based on authors’ clinical expertise [12]. Response options also included an “other” category with the opportunity for participants to provide written comments to both specific questions and the questionnaire in general. There was no word limit to the written-in comments.

### Data analysis

Descriptive statistics were used to characterize the sample and multiple-choice responses from the questionnaire. Responses were analyzed both combined across ICU stakeholders and based on specific roles grouped into: critical care physicians and critical care advance practice providers; bedside nurses; psychiatry physicians and psychiatry advance practice providers; occupational and physical therapists; and pharmacists. Qualitative data from open-ended responses were analyzed using content analysis. EDB generated codes based on latent content from review of the qualitative data across all open-ended comments. Comments were then double-coded by EDB and JBS, and Cohen’s kappa reliability coefficient indicated that interrater agreement was adequate ( κ = 0.70).

## Results

The survey was completed by 373 participants (response rate: 35.9%). The distribution of responses based on the stakeholder’s professional role is described in Table 1. The majority of survey participants routinely work in the medical ICU setting (37.8%), surgical ICU (34.3%), and a combination of both (20.1%); 7.8% of responders were psychiatry providers who do not predominately work in the ICU.

**Table 1:** Role distribution

Role	Survey Responses *N* = 373 (%)	Written Comments *N* = 74 (%)
Bedside nurse	207 (55.5)	26 (35.1)
Critical Care Staff Physician	45 (12.0)	17 (23.0)
Critical Care Fellow	23 (6.2)	4 (5.4)
Critical Care Advance Practice Provider	19 (5.1)	2 (2.7)
Psychiatry Staff Physician	2 (0.5)	0 (0.0)
Psychiatry Resident	10 (2.7)	6 (8.1)
Psychiatry Advance Practice Provider	2 (0.5)	0 (0.0)
Physical Therapist	26 (7.0)	4 (5.4)
Occupational Therapist	19 (5.1)	11 (14.9)
Pharmacist	20 (5.4)	4 (5.4)

### Patients’ experience in the ICU

90.5% of participants identified that they believed sedated patients in the ICU can hear speech, compared to 62.4% who believed sedated patients could process speech. 90.8% believed patients can remember portions of what is said to or around them; 89.7% believed patients can register, on some level, the emotional atmosphere in the room; and 82.7% believed patients can register, on some level, the emotional state of the provider interacting with them.

### Current and desired roles of psychiatry

47% of all non-psychiatry participants indicated they do not interact with psychiatry in their current professional role at all. Of all groups, bedside nurses have the least contact with psychiatry (53.9% selecting “never”). Others reported collaborating with psychiatry on a monthly (42.0%), weekly (7.8%), or daily (1.1%) basis. There was a significant difference between stakeholders in the amount of interaction with psychiatry, *F* = 8.53, *p* < 0.001, with critical care physicians and advance practice providers interacting more frequently with psychiatry than bedside nurses (*p* < 0.001) and OT/PT (*p* = 0.03).

Participants ranked the current primary role of psychiatry in the ICU as follows: assistance with management of mental health issues in the critically ill (37.6%), suicide risk assessments (23.1%), and treatment recommendations for delirium/agitation (17.7%); 8.3% selected more than one role. There was no difference among non-psychiatry stakeholders in terms of psychiatry’s perceived primary role as managing mental health issues (*p* = 0.16) or other areas (*p* = 0.17). There was a difference in perception of psychiatry’s primary role among stakeholders related to suicide risk assessments (*p* = 0.01) and delirium/agitation recommendations (*p* = 0.02), such that critical care physicians/advance practice providers were more likely to select suicide risk assessment (34.9%) and less likely to select delirium and agitation recommendations (7.0%) compared to other non-psychiatry stakeholder groups. In contrast to all other respondents, 50.0% of participants from psychiatry selected providing treatment recommendations for delirium/agitation as their current primary role in the ICU (*p* < 0.001). 13% of all participants felt that psychiatry currently does not have a role in the ICU with a significant difference among non-psychiatry stakeholders (*p* < 0.001); bedside nurses were overrepresented in this group.

Nonetheless, the idea of increased psychiatry presence in the ICU was well received, with 46.4% of responders welcoming an enhanced psychiatry presence and integration into the ICU team. There was a difference among non-psychiatry stakeholders (*p* < 0.001) with the majority of critical care physicians/advance practice providers expressing interest in integration (65.5%) and significantly fewer bedside nurses (17.7%) and occupational/physical therapists (11.1%) expressing interest.

### Benefit of psychiatry’s enhanced presence

Integration of psychiatry specifically was endorsed as potentially beneficial in preventing PICS by way of early psychological intervention in parallel with medical care (82.5%), identification of psychiatric and psychological factors impacting treatment (80.1%), assistance in pharmacologic management of refractory agitated delirium (75.2%), reduction of the patient’s psychological distress through communication (62.3%), reduction of learned helplessness by empowering the patient (58%), facilitation of communication between the patient and the primary team (52.8%), reduction in the incidence of delirium through additional communication and reorientation (51.2%), and facilitation of ventilator weaning by managing the patient’s anxiety (46.9%). Incorporation of psychiatry into the ICU team as a means of PICS prevention was considered superior to the initiation of antidepressant medication by the ICU team, utilization of ICU diaries, optimization of integrative medicine services, or no intervention by 83.4% of bedside nurses, 88.9% of occupational and physical therapists, 55.0% of pharmacists, 35.6% of critical care physicians and advance practice providers, and 35.7% of psychiatry physicians and advance practice providers. 11.3% of all participants chose “other” (see Table 2 for general comments arranged by theme). Critical care physicians and advance practice providers felt that psychiatry’s enhanced presence could be of most benefit in identifying psychological and psychiatric factors which may impact treatment (67.8%), while 9.2% thought there would be no benefit. Psychiatry participants anticipated the primary benefit to be assistance with pharmacologic management of refractory delirium (78.6%).

**Table 2:** Examples of comments, arranged by theme

Psychology (*N* = 19)
“It would be great to have psychology involved to potentially provide CBT and longitudinal care” – CC
“Assist in grief management”—PT
“Only concern is that psychiatry may lead to over medication of patients. Would appreciate greater level of nonpharmacologic interventions” –BN
Resource Limitations (*N* = 6)
“Not enough psych resources”—CC
[On barriers] “Resource allocation of such highly trained subspecialists in the critical care environment” – CC
“Availability”—PP
Education (*N* = 8)
“Assisting with bedside staff education on managing patients in psychological distress”— BN
“If Psychiatry can help educate the ICU staff, then ICU can better communicate with family” –PP
“Provide education to staff to improve our communications…”—OT
Burnout (*N* = 7)
“Decrease the burden on nurses dealing with depressed patients, especially the transplant or ECMO (pre-transplant) population that spends so much time in our ICU”—BN
“Addressing nursing moral distress in our ICU, it does not seem to be a singular issue but an issue with many nurses no one is talking or doing anything about” –BN
“Please bring a stronger psych presence to the ICU. At least for nurse burnout if for no other reason”—BN
Conflict and Expertise (*N* = 14)
“One more 'cook in the kitchen' will lead to more different 'voices' the family hears”—CC
“Conflicting opinions/goals, potential for delivery of mixed messages, potential splitting of provider teams depending on circumstances” —PP
“Psychiatrist are not trained nor comfortable in ICU” –CC
“Psychiatry does not understand ICU issues”—CC
Role Confusion (*N* = 9)
“Role confusion with nursing”—BN
“I would see their role as an adjunct not as a replacement to the critical care team’s presence in co-managing agitation/delirium etc..” CC
“These are all activities which OT has completed in the past and is within our scope of practice”—OT
“While I’m sure they could do many or all of these things, there are other members of the medical team and family support who could perform such tasks as well”—Pharmacist
Other (*N* = 11)
“We have multi-disciplinary rounds every Monday—Friday at 815. Maybe you could attend them on certain days to potentially get an idea if there are at risk patients on the unit”—BN
“The MICU team should be more mindful of what they say in the presence of the patient as the pt can hear”—BN
“I do think psych should be more involved in the ICU, but I do think that there are some cases where people will lose sight of the importance of prioritization”—BN

*CC* Critical care physician or advance practice provider, *OT* Occupational therapist, *PT* Physical therapist, *BN* Bedside nurse, *PP* Psychiatry physician or advance practice provider

Stakeholders felt that a more consistent psychiatry presence in the ICU could offer assistance to patients’ families by providing education on delirium and the patient’s mental status (84.6%). The majority also felt psychiatry could decrease the families’ psychological distress through communication (75.3%), educate patients’ families on strategies to help prevent delirium (69.1%), and facilitate communication with the critically ill (67.5%).

Regarding psychiatry presence benefitting ICU staff, reduction in provider burnout was selected by 86.0% of occupational and physical therapists and 82.0% of bedside nurses compared to 47.4% of pharmacists, 49.4% of critical care physicians and advance practice providers, and 50.0% of psychiatry responders. Bedside nurses offered more comments regarding the issue of burnout than any other group (see Table 2).

### Potential barriers to and drawbacks of enhanced psychiatry’s presence

Participants were most concerned that an enhanced presence of psychiatry in the ICU could cause distress to the patient and/or their family members (40.1%). 1.4% of all participants indicated that they would feel stigmatized by psychiatry’s presence at bedside if they or their loved ones were in the ICU, while another 7.0% indicated they would prefer only medical treatment and would deal with stressors on their own.

Other foreseeable potential barriers were the disruption of the ICU team’s work flow by psychiatry visiting with the patient (30.5%), and the distraction from medical issues by focusing on psychosocial matters (30.8%). 30.8% did not identify any foreseeable barriers.

### Qualitative content analysis

Fifty participants provided 74 total comments with 1488 words. Comments were provided by participants from each group, with most comments provided by bedside nurses (35.1%) and critical care physicians/advance practice providers (31.1%). Six prominent themes were identified: the benefit of psychology over psychiatry; concern for conflict and expertise; concern for role confusion; psychiatry’s role in providing education in the ICU; burnout; and resource limitations. Representative comments, abridged and arranged by theme, are provided in Table 2.

### Psychology versus psychiatry

Nineteen comments reflected the desire to increase psychological support for critically ill patients. These referenced enhancing opportunities to process difficult news, providing skills to cope with illness, identifying anxiety, and targeting symptoms through non-pharmacologic interventions, specifically Cognitive Behavioral Therapy. Critical care physicians and advance practice providers, psychiatry physicians and advance practice providers and pharmacists identified these goals as likely best addressed by psychology rather than psychiatry.

### Conflict and expertise

The possibility of conflict was referenced by 11 participants across all groups. Contrary opinions regarding pharmacological treatment and communication of inconsistent messages to patients and/or patients’ families were identified as the possible sources of such conflict. In addition, 3 comments from critical care physicians also expressed concern over psychiatry’s skill and/or comfort in the ICU.

### Role confusion

Nine comments from non-psychiatry participants suggested that many of the proposed potentially beneficial interventions fall within the scope of their own practice, and that the addition of psychiatry may lead to confusion among team members and burden the patient by introducing yet another provider.

### Education

Eight comments identified psychiatry’s desired role in the ICU as providing education and/or guidance to the current ICU team on how to offer support to patients and families and navigate challenging conversations.

### Burnout

Seven comments underscored burnout prevention as a possible benefit of psychiatry’s enhanced presence in the ICU. All but one were written by bedside nurses, who highlighted the emotional burden of working closely with very ill and/or dying patients, and the negative impact burnout has on one’s capacity to provide emotional support to patients and their families.

### Resource limitations

Six comments, three from critical care physicians/advance care providers, two from psychiatry physicians/advance care providers and one from a bedside nurse, highlighted limited psychiatric resources as a potential barrier to psychiatry’s enhanced presence in the ICU.

### Other

The remaining 11 comments did not fit a particular theme and were labeled as “other”.

## Discussion

To the best of our knowledge, this study is the first to report feedback on increasing psychiatry’s presence in the ICU, potential areas of benefit, including the prevention of PICS, and drawbacks. In the conventional model of psychiatry consultation, providers in the ICU are responsible for identifying psychiatric concerns and requesting psychiatry involvement. However, prior studies have identified a much lower rate of psychiatry involvement in the ICU than other hospital settings [11], findings consistent with the nearly half of ICU stakeholders who reported no contact with psychiatry in the current study. This may be due to the fact that many critically ill patients are in poor condition to communicate. However, over 90% of survey responders thought that sedated patients can hear speech and remember portions of what was being said around them; over two thirds also thought that sedated patients could process speech, potentially expanding the therapeutic window for an intervention. In addition, psychiatry consultation may be limited due to poor identification of mental health concerns. For example, agreement rates between psychiatric diagnoses (including delirium) made by inpatient primary teams and the consulting psychiatrist have been described as low as 41%, and studies suggest that delirium may be frequently missed [13] or misinterpreted as depression, anxiety, and bipolar disorder [14]. Discrepancy in psychiatric diagnosis is concerning as delirium has been implicated (although inconsistently) as a risk factor for the development of PICS, including cognitive and mental health impairments [15].

Based on research in other hospital settings, limited ICU psychiatry consultations may also be indicative of historic stigma against mental health services, inadequate rapport between other providers and the psychiatrist, and belief that another provider may address concerns without psychiatry involvement [12]. Consistent with these previously identified barriers to psychiatric consultation, qualitative findings indicated concern about potential role confusion between psychiatry and bedside nurses, OT and pharmacy. Roughly 1% of the study responders felt that psychiatric involvement would make patients feel stigmatized. While a third of responders foresaw no barriers to psychiatry’s presence, over a third were concerned that it may interrupt team work flow or cause family distress. Other comments reflect a concern of potential conflict in pharmacologic management of delirium and agitation. Nonetheless, over 90% indicated that they would like psychiatry available to monitor for depression, anxiety or other symptoms that warrant intervention, indicating that the concerns were minimal overall.

Most ICU stakeholders appreciated the need for increased psychological support for patients in the ICU; however, opinions on psychiatry’s current and desired roles, as well as on potential benefits and drawbacks of increasing its involvement, varied across provider groups. Those with greater professional familiarity with psychiatry (critical care physicians, advance practice providers, and pharmacists), as well as psychiatry responders themselves, questioned the feasibility of integrating a psychiatrist into the ICU team, referencing the number of ICUs and scarcity of psychiatric resources. Those with less familiarity with psychiatry, though generally with more actual patient contact time (bedside nurses, occupational and physical therapists) had the lowest interest in psychiatry integration among the non-psychiatry stakeholder groups. Comments from these groups expressed stronger interest in having a qualified mental health professional rather than psychiatry in particular becoming a part of the team. Comments included multiple suggestions of cognitive behavioral therapy and “a consistent psychologist to see the patient on a daily basis” as well as concern of “overmedicating the patient; would appreciate greater level of nonpharmacologic interventions”.

Presence of mental health professionals in the ICU is not a novel idea; it has been implemented in centers around Europe since the early 2000s. In the United Kingdom, the 2019 Guidelines for the Provision of Intensive Care Services outline specific recommendations for the role of psychologists in the ICU, which are consistent with the desired roles identified by our study’s participants. These recommendations include: supervision of delirium screenings; education of patients, patients’ families and ICU staff on the possible psychological impact of medical interventions and the ICU environment in general; delivery of psychological assessments and interventions to those at risk; administration of psychological support to family members; and development of support programs for ICU staff to promote well-being and reduce burnout [16]. Despite this, presence of psychology in the ICU setting remains scarce with only 17% of responding units offering psychological services [17]. In the United States, a survey of the American Psychological Association list serve’s practicing psychologists identified 51 individuals who provide cognitive assessments, family support, education, behavioral management, psychotherapy, and/or suicide risk assessments in critical care settings [18]. Limited outcome data is available to date, however. The addition of a clinical psychologist to a trauma ICU team significantly lowered PTSD rates compared to historical controls, and [19] the use of positive suggestion by trained psychologists was associated with less need for hypnotics and analgesics, shorter duration of mechanical ventilation, and shorter duration of the ICU length of stay [20–22].

Efforts to expand current knowledge regarding the benefit of mental health providers (both psychologists and psychiatrists) are underway. An emerging collaborative approach in delirium management [23] has been documented; a current trial is examining the effectiveness of therapeutic suggestion on mental and cognitive health outcomes provided to ICU patients by trained doulas, with collaboration from psychiatry [24]. Psychologists successfully applied therapeutic suggestion to reduce anxiety of patients on non-invasive ventilation [25]. An embedded ICU rehabilitation psychologist was successful providing consultations to patients with prolonged hospitalization, which led to a reduction in anxiety ratings [26]. Many of these benefits were similarly endorsed as potential positives of greater psychiatry involvement by the majority of respondents in the current study as well.

Another potential benefit of increasing psychiatry’s presence in the ICU identified by survey responders was the reduction of staff burnout. Staff well-being has been studied in relation to patient safety [27] and satisfaction outcomes [28]. To the best of our knowledge, there have been no studies examining the effects of staff burnout on patients’ mental health outcomes, but it is conceivable that such a relationship may exist: over 80% of our survey responders thought that sedated patients could register, on some level, the emotional state of the provider interacting with them. Burnout is characterized by emotional exhaustion, cynicism, and sense of inefficacy; [29] it has been recognized as highly prevalent among ICU clinicians, [28, 29] especially nurses [30, 31]. Critically ill patients experience alterations in thought process with markedly diminished ability for abstraction and increased susceptibility to suggestion without critical appraisal [32, 33]. Staff who are experiencing emotional exhaustion themselves may be able to offer fewer messages of comfort and healing to patients, and perhaps even, unintentionally, share messages of doubt, distrust, and pessimism [34, 35]. Although speculative, addressing staff well-being may have positive effects on mental health outcomes in ICU survivors. Psychiatry’s potential role in ICU staff well-being is an area of active research under the current pandemic conditions: University of Minnesota is trialing a rapidly deployable Psychological Resilience Intervention, coordinated by the Departments of Anesthesiology and Psychiatry & Behavioral Science, intended to provide resilience-promoting strategies [36].

This study has several limitations. Data were collected from one specific large institution and ICU provider perspectives may not generalize to other hospitals in the United States or aboard. Multiple-choice options may have missed choices that would better identify stakeholder perspectives and may have forced participants to conform their opinion to best fit an available response. Attempts to mitigate this included the option of selecting “other” and providing room for open ended responses, which participants did utilize. Questions and responses did not differentiate between psychiatry and psychology, as the institution where data were collected did not have an inpatient psychology team and, multiple choice responses were created to include roles of both. Psychiatry providers, especially psychiatry staff physicians, were underrepresented in this sample. This may be due to the relatively low number of psychiatry staff on the inpatient consultation service. In general, however, survey response rate and the number of comments is considered to adequately reflect the perceptions of this institution’s ICU stakeholders and is consistent with prior studies. Diversity of critical care stakeholders surveyed adds to the generalizability of the findings.

## Conclusion

Psychiatric illness in the critically ill is higher than in the general population, and is a poor prognostic factor for mental health outcomes among survivors. Those who work closely with critically ill patients suggest that increased psychological support in the ICU could be beneficial. By contrast, psychiatry’s involvement is seen to be limited, consistent with study hypothesis. Limited current involvement is perhaps driven by varying perceptions of what psychiatry’s role is, or should be, or a perceived barrier of limited staff resources. While many ICU providers, especially critical care physicians and advanced practice providers expressed interest in increased psychiatry involvement and even integration into the ICU team, concerns regarding patients’ responses to psychiatry’s involvement and concern for “too many cooks in the kitchen” were highlighted as potential drawbacks. These findings may inform efforts to refine multidisciplinary collaborations for the benefit of ICU patients. Clarification of psychiatric versus psychological interventions in the ICU also warrant further study.

## Supplementary Information

**Additional file 1.**

## Data Availability

The datasets used and/or analyzed during the current study available from the corresponding author on reasonable request.
