# Supplementary material for: Psychiatry’s role in the prevention of post-intensive care mental health impairment: stakeholder survey
Source: BMC Psychiatry. 2022 Mar 18;22:198. doi: 10.1186/s12888-022-03855-w (PMC8933991; doi:10.1186/s12888-022-03855-w)
Supplement: Supplementary file 1 — Additional file 1. [file 12888_2022_3855_MOESM1_ESM.docx]

Supplementary Table 1, Additional File 1

Psychiatry in the ICU Survey

1. What is your role? Critical Care Consultant

Critical Care NP/PA Critical Care Fellow Bedside Nurse


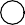

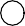

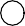

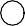

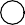

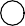

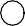

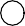

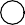

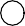


OT PT

Psychiatry Consultant Psychiatry Resident Psychiatry NP/PA Pharmacist

1. Which ICU setting do you typically work in? Medical ICU Surgical ICU

Medical and surgical ICU


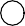

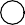

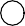

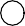


Most of my work is not in the ICU

1. How frequently do you interact with psychiatry in your I am part of the psychiatry team; does not apply professional role? Not at all

On a monthly basis On a weekly basis On a daily basis


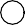

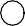

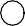

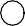

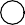


1. From your experience, what is the primary role of None

psychiatry in the ICU currently? Treatment recommendations for delirium/agitation Suicide risk assessments


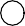

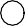

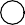

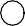


Assistance with management of mental health issues in the critically ill


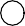
 Other:

If you selected "other", please specify:

1. Given that many ICU patients suffer from

post-intensive care syndrome or PICS (new or worsening impairments in mental, cognitive and physical domains following resolution of critical illness), current

practice involves ABCDEF bundle/early occupational therapy to prevent delirium/subsequent cognitive problems and early physical therapy to prevent

ICU-acquired weakness. What approach in your opinion would be the best to address prevention of post-ICU mental health problems affecting over 1/3 of patients?

The medical team should start patients on an antidepressant in the ICU

Nurses should utilize ICU diaries on all patients Psychiatry should become a part of the ICU team and work with at-risk patients on a daily basis Integrative medicine should become a part of the ICU team and offer pet therapy/massage/other interventions to patients at risk on a daily basis No prevention is feasible; this should be addressed in the post-ICU clinic

Other:

If you selected "other", please specify:

1. Do you think that sedated patients can... (check all that apply)

Hear speech Process speech

Remember portions of what is said to them or around them

Register, at some level, the emotional atmosphere in the room, e.g., anxiety, confidence, tension, compassion...

Register, at some level, the emotional state of the provider interacting with them

Don't know

1. What benefit could a more consistent psychiatry presence in the ICU offer to the patient? (check all that apply)

None

Decrease incidence of delirium through additional communication and reorientation

Assist in pharmacologic management of refractory agitated delirium

Decrease patient's psychological distress through communication

Facilitate communication between patient and the primary team

Decrease learned helplessness by empowering the patient

Provide early psychological support in parallel with medical care

Facilitate ventilator weaning by managing patient's anxiety

Identify psychiatric and psychological factors which may impact treatment

Other:

If you selected "other", please specify:

1. What benefit could a more consistent psychiatry presence in the ICU offer to the family? (check all that apply)

None

Decrease family's psychological distress through communication

Help family understand the patient's mental state/delirium better

Teach family specifics about communicating with the critically ill

Teach family strategies to help the patient (including bringing familiar objects from home) Facilitate communication between family and the primary team

Other:

If you selected "other", please specify:

1. What benefit could a more consistent psychiatry presence in the ICU offer to the staff? (check all that apply)

None

Assist with management of centrally acting medications

Facilitate communication with patient and family Reduce provider burnout by helping to process difficult interactions or situations

Other:

If you selected "other", please specify:

1. What barriers could you foresee with a more consistent psychiatry presence in the ICU? (check all that apply)

None

Patient or family distress at the presence of psychiatry

Interruption of the team's work flow by psychiatry visiting with the patient

Distraction from medical issues at hand due to focus on psychosocial matters

Other:

If you selected "other", please specify:

1. If you or your loved one were critically ill, would
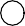
 Yes, I would like psychological aspects of my you like psychiatry at bedside to attend to your critical illness to be addressed along the needs? physical ones


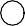
 Yes, I would like psychiatry available to monitor for depression, anxiety or other symptoms that warrant intervention


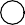
 No, I would prefer only medical treatment, and would find a way to deal with stressors on my own afterwards


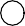
 No, I would feel stigmatized by the idea of having psychiatry at bedside


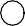
 Other:

If you selected "other", please specify:

1. Overall, what do you think of the current extent of They are too involved already psychiatry involvement in the ICU? Just right

Not enough. I would like their involvement more often


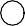

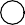

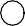


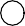
 I think everyone would benefit most if psychiatry could become a permanent member of the ICU team like pharmacy, PT, OT

Other comments on psychiatry presence in the ICU:
